# Investigating the anti-obesity potential of *Nelumbo nucifera* leaf bioactive compounds through machine learning and computational biology methods

**DOI:** 10.3389/fphar.2024.1500865

**Published:** 2024-12-18

**Authors:** Hongyun Huang, Chengyu Liu, Can Cao, Moxi Chen, Ruyin Li, Jianchun Yu

**Affiliations:** Department of General Surgery, Peking Union Medical College Hospital, Peking Union Medical College and Chinese Academy of Medical Sciences, Beijing, China

**Keywords:** *Nelumbo nucifera* leaves, network pharmacology, machine learning, obesity, molecular dynamics simulation

## Abstract

Obesity, a growing global health concern, is linked to severe ailments such as cardiovascular diseases, type 2 diabetes, cancer, and neuropsychiatric disorders. Conventional pharmacological treatments often have significant side effects, highlighting the need for safer alternatives. Traditional Chinese Medicine (TCM) offers potential solutions, with plant extracts like those from *Nelumbo nucifera* leaves showing promise due to their historical use and minimal side effects. This study employs a comprehensive computational biology approach to explore the anti-obesity effects of *Nelumbo nucifera* Leaf Bioactive Compounds. Sixteen active compounds from *Nelumbo nucifera* leaves were screened using the Traditional Chinese Medicine Systems Pharmacology Database (TCMSP). Clustering analysis identified three representative molecules, and network pharmacology pinpointed PPARG as a common target gene. Molecular docking and machine learning models were used for inhibitors screening, and molecular dynamics simulations were futher used to investigate the inhibitory effects and mechanisms of these molecules on PPARG. Subsequent cellular assays confirmed the ability of Sitogluside to reduce lipid accumulation and triglyceride levels in 3T3-L1 cells, underscoring its potential as an effective and safer obesity treatment. Our findings provide a molecular basis for the anti-obesity properties of *Nelumbo nucifera* Leaf Bioactive Compounds and pave the way for developing new, effective, and safer obesity treatments.

## 1 Introduction

Obesity is a prevalent and escalating health issue, closely associated with severe conditions such as cardiovascular diseases, type 2 diabetes, cancer, and neuropsychiatric disorders (Lopez-Jimenez et al., 2022; Khan and Hegde, 2020; Martins, 2013). Globally, obesity rates have more than tripled since 1975, with recent data indicating that over 650 million adults are obese, contributing significantly to the global burden of non-communicable diseases and healthcare costs (Mohajan and Mohajan, 2023). This condition not only contributes significantly to the global burden of non-communicable diseases but also imposes a heavy psychological and physical toll on individuals, often leading to depression, anxiety, reduced mobility, and diminished quality of life. Additionally, obesity is associated with increased healthcare costs and reduced productivity, thereby impacting both personal wellbeing and socioeconomic stability. The urgent need for effective pharmacological interventions is undeniable. Current treatments, including phentermine (Smith et al., 2013), fluoxetine (Wise, 1992), orlistat (Ballinger and Peikin, 2002), sibutramine (Padwal and Majumdar, 2007), and rimonabant (Curioni and André, 2006), each with distinct mechanisms of action. Phentermine and sibutramine primarily work by suppressing appetite through central nervous system stimulation, while orlistat inhibits lipid absorption in the gastrointestinal tract. Fluoxetine and rimonabant modulate neurotransmitter activity to influence appetite and satiety. Despite their efficacy, these pharmacological interventions are often associated with adverse effects, including nausea, dizziness, insomnia, and gastrointestinal discomfort, which limit their long-term use for obesity management (Velazquez and Apovian, 2018; Patel, 2015). These challenges, combined with the difficulty of maintaining a healthy lifestyle and the invasiveness of surgery, have sparked interest in natural therapies (Acosta et al., 2014). Traditional Chinese medicine, known for its milder side effects, has emerged as a promising alternative (Qi et al., 2015).

Plant-derived compounds have attracted considerable attention for their potential role in obesity management due to their multifaceted mechanisms of action, lower toxicity, and diverse bioactive components. These compounds, which include polyphenols, flavonoids, alkaloids, and terpenes, are known to interact with key metabolic pathways, influence lipid metabolism, and exhibit antioxidant and anti-inflammatory properties that can address various obesity-related health issues. For instance, polyphenols such as curcumin, resveratrol, and proanthocyanidins have demonstrated the ability to modulate adipocyte differentiation, reduce lipid accumulation, and improve insulin sensitivity, making them valuable in the fight against obesity (Sergent et al., 2012; Boccellino and D’Angelo, 2020). Additionally, Garcinia cambogia extract, which contains hydroxycitric acid, is widely used for weight management without toxic effects (Onakpoya et al., 2011; Semwal et al., 2015).

One particularly promising natural therapy is the use of *Nelumbo nucifera* leaves (*Nelumbo nucifera*) (Wang et al., 2021), which have been employed for their anti-obesity properties since the Ming Dynasty in China over 1,000 years ago (Fan et al., 2021; Zheng et al., 2010). Initially documented in “The Key to Diagnosis and Treatment,” *Nelumbo nucifera* leaves have recently gained popularity in China as tea and dietary supplements for weight loss and lipid reduction (Allison et al., 2001). Additionally, Diospyros (D.) *Nelumbo nucifera*, known for its sedative, anti-diabetic, antiseptic, and anti-tumor properties, has fruits and roots that exhibit anti-proliferative and cytotoxic effects on various cancer cell lines (Rauf et al., 2017). *Nelumbo nucifera* leaves have also been used to alleviate muscle and joint pain (Sridhar and Bhat, 2007). However, the anti-obesity potential of *Nelumbo nucifera* leaves remains underexplored (Liu et al., 2023). While synthetic anti-obesity drugs target specific pathways such as appetite suppression and lipid absorption, natural compounds like polyphenols and alkaloids provide a multi-target approach with generally milder side effects. This distinction highlights the complementary potential of plant-derived compounds, which can influence similar metabolic pathways in a holistic manner, offering potentially safer and more sustainable solutions for obesity management.

In recent years, machine learning has revolutionized various domains of science, and its integration into cheminformatics has significantly transformed drug discovery and development processes. Cheminformatics involves the application of computational techniques to solve chemical problems, particularly in drug design and toxicology. Machine learning, a subset of artificial intelligence, has been widely used in cheminformatics to predict molecular properties, bioactivities, and toxicity profiles, enabling the identification of potential drug candidates more efficiently. By learning from large datasets of chemical and biological information, machine learning models can predict the interactions between small molecules and biological targets, facilitating virtual screening, molecular docking, and drug repurposing efforts. These advancements are crucial in identifying novel therapeutic agents, including those derived from natural products, such as Traditional Chinese Medicine (TCM) (Nestler, 2002).

**FIGURE 1:**
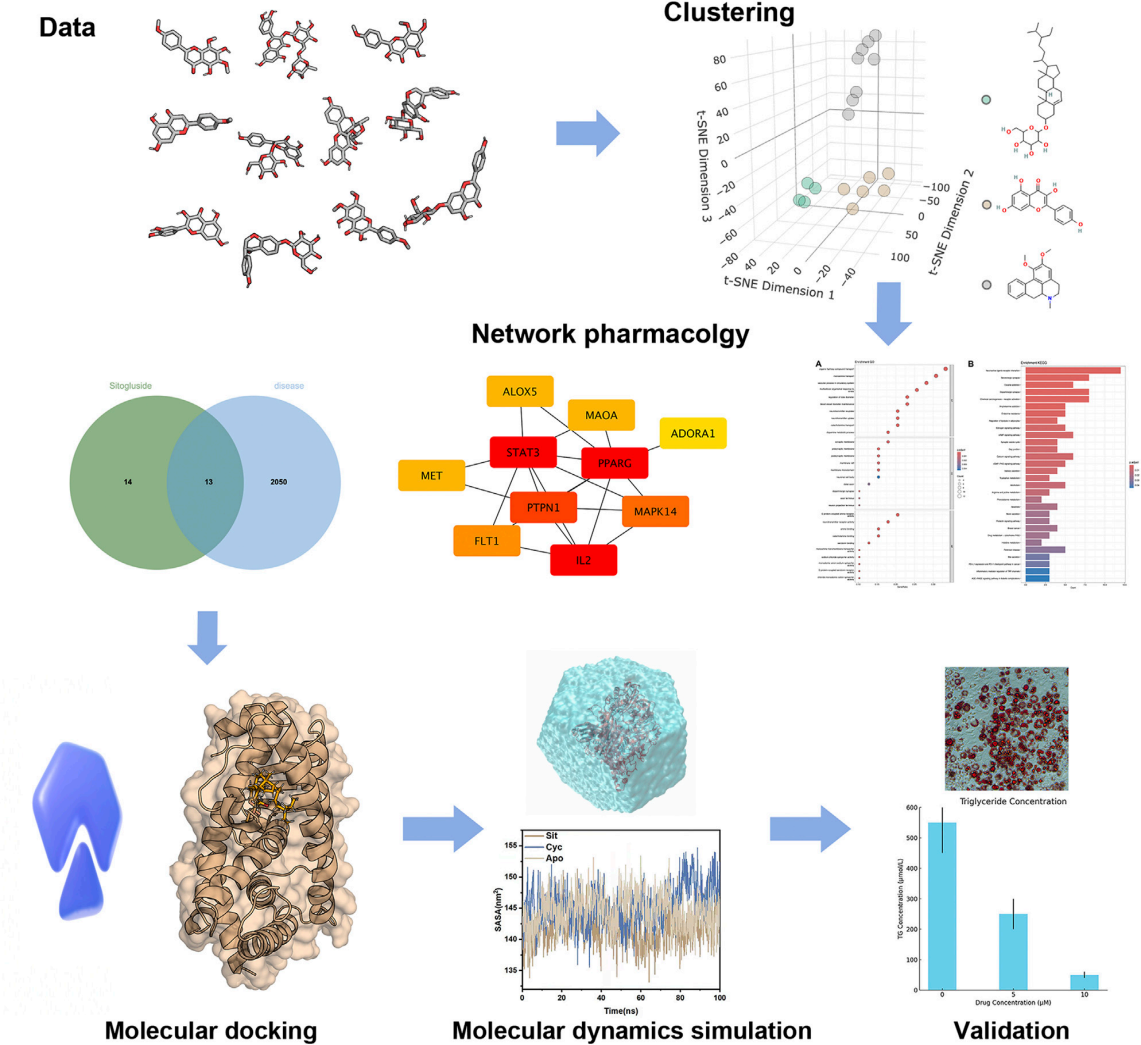
The work flow of our study.

This research adopts a comprehensive approach utilizing various computational biology methods. Sixteen highly absorbable small molecules from *Nelumbo nucifera* leaves were screened in the Traditional Chinese Medicine Systems Pharmacology Database (TCMSP) (Ru et al., 2014). Clustering analysis identified three representative molecules, and network pharmacology analysis revealed PPARG as their common target gene. Subsequent molecular docking examined the inhibitory effects of these molecules on PPARG, and molecular dynamics simulations explored the underlying mechanisms. By integrating these advanced computational techniques, the study aims to elucidate the molecular basis of the anti-obesity effects of *Nelumbo nucifera* Leaf Bioactive Compounds, potentially paving the way for the development of new, effective, and safer obesity treatments. Our workflow was shown in Figure 1.

## 2 Materials and methods

### 2.1 Clustering analysis of small molecule data

Using the Traditional Chinese Medicine Systems Pharmacology Database and Analysis Platform (TCMSP, https://tcmsp-e.com/), we screened all active components of *Nelumbo nucifera* leaves based on criteria of oral bioavailability (OB) ≥ 20% (Veber et al., 2002) and drug-likeness (DL) ≥ 0.18 (Ursu et al., 2011). To analyze the clustering of small molecule similarities, we utilized molecular fingerprints, dimensionality reduction, clustering, and 3D visualization techniques. Specifically, Morgan fingerprints were computed for each compound using RDKit (Landrum, 2013), with a radius of 2 and 2048 bits. SMILES strings were converted to molecular objects for fingerprint generation, excluding invalid ones, and duplicates were removed to ensure uniqueness in the dataset. We applied t-SNE (Van der Maaten and Hinton, 2008) with a perplexity of 30 to reduce the fingerprint data to three dimensions. Hierarchical clustering using Ward’s method was then performed on the t-SNE results (Van der Maaten and Hinton, 2008), setting the number of clusters to three. Each compound was assigned a cluster label, and within each cluster, we calculated a similarity matrix using the Tanimoto coefficient (Bajusz et al., 2015). The molecule with the highest average similarity within each cluster was identified as the representative. A 3D scatter plot was created using Plotly, coloring each compound by cluster assignment and distinctly marking representative molecules, enabling intuitive exploration of small molecule similarities and relationships (He et al., 2022).

### 2.2 Prediction and analysis of potential obesity targets and *Nelumbo nucifera* leaf component interactions

In this study, potential target genes related to obesity were initially identified from four authoritative databases: DisGeNET (Piñero et al., 2016), GeneCards (Safran et al., 2010), PharmGKB (Hewett et al., 2002), and UniProt (UniProt Consortium, 2015). By comprehensively comparing these databases and removing duplicates, we screened a total of 2,063 potential target genes for further analysis. To predict possible interactions between the active components of *Nelumbo nucifera* leaves and these obesity target genes, we utilized databases and tools such as SEA, SuperPred (Gallo et al., 2022), and SwissTargetPrediction (Daina et al., 2019). These platforms enabled us to perform an in-depth prediction of intersection target genes for the active components found in *Nelumbo nucifera* leaves.

### 2.3 Construction and analysis of the Protein-Protein Interaction network

To explore the potential interactions among key target proteins, we constructed a comprehensive Protein-Protein Interaction (PPI) (Athanasios et al., 2017) network using the STRING database (Wagner and Fischer, 1974), a prominent bioinformatics tool. This network was visualized with the latest version of Cytoscape software (Kohl et al., 2011). We conducted a thorough analysis of the network’s topological features using advanced analytical tools within Cytoscape, identifying ten hub genes that play a crucial role in the disease mechanism.

### 2.4 Comprehensive enrichment analysis of GO and KEGG pathways

We performed enrichment analyses of Gene Ontology (GO) (Harris et al., 2004) and Kyoto Encyclopedia of Genes and Genomes (KEGG) (Kanehisa and Goto, 2000) pathways using R packages including clusterProfiler (Yu et al., 2012), org. Hs.eg.db (Carlson et al., 2019), and pathview. Gene lists were converted to Entrez IDs and analyzed for enrichment using enrichGO and enrichKEGG with *p*-value and q-value cutoffs of 0.01 and 0.05, respectively. Results were visualized with dot plots and bar plots, highlighting significant terms and pathways. Additionally, pathview was used to map genes onto KEGG pathways for detailed visualization (Wang et al., 2024).

### 2.5 Batch molecular docking of active components

A receptor protein model based on PPARG (PDB ID: 8WFE) was constructed, encompassing 268 residues. The crystal structure was the latest structure available and was determined using the gold standard X-ray diffraction method, with a relatively high resolution of 2.20 Å. Additionally, it is a human-derived protein, and it is free from the conformational influences of other ligands or bound proteins, thus more closely representing the natural conformation. Therefore, we selected this structure. Homology modeling was performed with MODELER 10.1 to include missing residues and domains. Small molecules, initially in SMILES format, were converted to pdbqt format using Open Babel (O’Boyle et al., 2011). Molecular docking was then carried out using AutoDock Vina 1.2.0 (Eberhardt et al., 2021). The docking box was set to dimensions of 29.25 Å × 34.5 Å × 21.0 Å with center coordinates at x = −7.574, y = 10.801, z = 138.847. The docking site was chosen because it was described as the binding site for PPARG inhibitors and inverse agonists (Irwin et al., 2022; Nolte et al., 1998). Batch docking of 18 small molecules with their respective target proteins was conducted. This methodology enabled a detailed evaluation of potential inhibitors by analyzing their predicted interaction strengths with the target enzyme (Song et al., 2024). We also used the R357A PPARG mutant (PDB ID: 4O8F) and the V290M PPARG mutant (PDB ID: 4OJ4) and conducted molecular docking at the same site with a consistent method.

### 2.6 Machine learning

In this study, machine learning models were developed to predict the activity of PPARG inhibitors based on different molecular fingerprints. Molecular data for PPARG inhibitors were collected from publicly available databases, including CHEMBL (Gaulton et al., 2012) and PUBCHEM (Kim et al., 2019) and were represented as SMILES (Simplified Molecular Input Line Entry System) strings (Weininger, 1988). There were in total 7899 PPARG inhibitors, and for data balancing, we selected 7,900 inactive molecules. These SMILES strings were then converted into various molecular fingerprints to capture different structural and chemical features. The dataset was divided into training and test sets in an 8/2 ratio using the train_test_split function from Scikit-Learn, ensuring that both sets maintained a representative distribution of the data.

Five types of molecular fingerprints were generated: MACCS Keys (Jow et al., 1990), Morgan (Zhong and Guan, 2023), RDKit (Qiao et al., 2021), Topological Torsion (Nilakantan et al., 1987), and Atom Pairs fingerprints (Awale et al., 2015). MACCS Keys fingerprints were generated using RDKit’s GetMACCSKeysFingerprint function, which provides a 166-bit vector representation of predefined structural keys. Morgan fingerprints, analogous to Extended Connectivity Fingerprints (ECFP), were created using GetMorganFingerprintAsBitVect with a radius of 2 and 1,024 bits to capture circular substructures. RDKit fingerprints, generated with RDKFingerprint, encoded molecular substructures based on paths of bonded atoms. Topological Torsion fingerprints, produced by GetHashedTopologicalTorsionFingerprintAsBitVect, represented topological torsions within the molecular structure. Finally, Atom Pairs fingerprints, created using GetHashedAtomPairFingerprintAsBitVect, captured the relationship between atom pairs in terms of their topological distances.

Two machine learning algorithms, Random Forest (RF) (Belgiu and Drăguţ, 2016) and Extreme Gradient Boosting (XGBoost) (Sheridan et al., 2016), were employed for model development. The Random Forest model, implemented using Scikit-Learn’s RandomForestClassifier, is an ensemble method based on decision trees. Hyperparameters for the RF model, including the number of trees, maximum tree depth, minimum samples per split, minimum samples per leaf, and bootstrap sampling, were optimized using GridSearchCV. XGBoost, implemented with the XGBClassifier from the XGBoost library, is a gradient boosting algorithm. Key hyperparameters such as the number of estimators, maximum tree depth, learning rate, subsample ratio, and column sampling by tree were similarly optimized. Both models were trained using 5-fold cross-validation with KFold to ensure robust evaluation and hyperparameter optimization. The Area Under the Receiver Operating Characteristic Curve (AUC-ROC) was used as the primary scoring metric for model selection.

The performance of the trained models was evaluated on the test set using several metrics. These included AUC-ROC, which provides a comprehensive measure of model performance across all classification thresholds, sensitivity (SE) to assess the true positive rate, specificity (SP) to evaluate the true negative rate, and the Matthews Correlation Coefficient (MCC) to measure the overall quality of binary classifications. These metrics collectively provided a thorough assessment of the models’ ability to predict RANKL inhibitory activity based on different molecular fingerprints.

### 2.7 Molecular dynamics simulations

We conducted molecular dynamics (MD) simulations (Hollingsworth and Dror, 2018) on two key molecules, Cycloartenol and Sitogluside, using the PMEMD module of Amber 22 (Case et al., 2022) with CUDA acceleration. Each system underwent 100 ns MD simulations. Hydrogen bonds were constrained using the SHAKE algorithm (Elber et al., 2011), and electrostatic interactions were managed with the Particle Mesh Ewald (PME) method (He et al., 2024), with an 8 Å cutoff. Following initial system construction, atomic clashes were resolved through 500 steps of steepest descent and conjugate gradient minimization. Systems were then heated from 0 K to 300 K over 50 ps, followed by density equilibration and constant pressure operations at 300 K for 500 ps in the NPT ensemble. Once the systems stabilized, three 100 ns MD simulations were run, with data recorded every 1 fs using a 2 fs time step and a Langevin thermostat with a 1 ps collision frequency. Data storage occurred every 2 ps, resulting in 2,000 frames for analysis. Trajectory analysis was performed using the CPPTRAJ (Roe et al., 2013) module of Amber 22, assessing RMSD, RMSF, radius of gyration (Rg), and solvent-accessible surface area (SASA). K-means clustering within CPPTRAJ produced 10 representative structures. The binding free energy differences of protein-ligand complexes were estimated using MM-PBSA (Genheden and Ryde, 2015) calculations from 500 snapshots of the final trajectory. This method reduces errors related to covalent energy and is frequently used alongside MM-GBSA to predict binding free energies in a continuum solvent model.

#### 2.7.1 Secondary structure analysis

We analyzed the protein’s secondary structure using AMBER tools. First, the protein structures were aligned, and the secondary structure of each residue for every frame was outputted into a data file. The file was then modified to correct initial settings and enhance visualization by adding lines to set parameters for grid settings, color palettes, and axis labels. These adjustments allowed for accurate plotting of the data. The modifications included mapping the secondary structure data over time and adjusting the output settings to generate a detailed visual representation. Finally, the modified script was executed to produce a comprehensive plot of the protein’s secondary structure, enabling thorough analysis and interpretation of structural changes over time.

#### 2.7.2 Covariance matrix analysis

To analyze the covariance matrix of the protein, we utilized the Bio3D package (Grant et al., 2021) in R. Initially, the protein structure file (PDB) and the trajectory file (DCD) were loaded into the environment. The Bio3D package was then employed to facilitate the analysis. We selected the C-alpha atoms for the analysis to focus on the protein’s backbone. The trajectories were fitted to the reference structure to ensure proper alignment by aligning the C-alpha atoms in both the fixed and mobile structures. Subsequently, the covariance matrix was computed using the aligned coordinates of the C-alpha atoms. Finally, the covariance matrix was visualized to interpret the correlations between the movements of different parts of the protein.

#### 2.7.3 Principal component analysis

We conducted a Principal component analysis (PCA) (Abdi and Williams, 2010) using AMBER tools. First, the root mean square deviation (RMSD) was calculated for the initial structure (residues 1–269, excluding hydrogen atoms). An average structure was generated, and its RMSD was recalculated against the initial structure. A covariance matrix for the same residues was then constructed. Principal component analysis was performed by diagonalizing the covariance matrix, and the first two eigenvectors were extracted. These eigenvectors were used to project the conformational changes of the protein, providing insights into the dominant motions within the molecular dynamics simulation.

### 2.8 Validation of sitogluside on adipogenesis in 3T3-L1 cells

The murine embryonic fibroblast cell line, 3T3-L1, was procured from the National Infrastructure of Cell Line Resource (China Infrastructure of Cell Line Resource). Dulbecco’s Modified Eagle’s Medium (DMEM) was purchased from Gibco. Sitogluside was obtained from TargetMol. The triglyceride (TG) assay kit was sourced from Applygen Technologies. Fetal calf serum (FCS) was supplied by Lablead. The modified Oil Red O staining kit was procured from Beyotime. Isobutylmethylxanthine (IBMX) was acquired from Sigma-Aldrich, Dexamethasone from MedChemExpress, and Insulin from Lablead.

Cell Culture and Treatment: 3T3-L1 preadipocytes were maintained in Dulbecco’s Modified Eagle’s Medium (DMEM) supplemented with 10% fetal calf serum, at 37°C in a 5% CO2 atmosphere. Differentiation was induced when cells reached confluence, using a differentiation medium containing 1 µM Dexamethasone, 0.5 mM IBMX, and 10 μg/mL Insulin with 10% FCS for 48 h. Thereafter, the medium was replaced with DMEM containing only 10 μg/mL Insulin and the cells were cultured for an additional 5–7 days, with the medium refreshed every 2 days.

Sitogluside Treatment: Post-differentiation, 3T3-L1 adipocytes were treated with Sitogluside at concentrations of 0 μM, 5 μM, and 10 µM. The treatments were administered in DMEM throughout the experiment to evaluate their effects on lipid accumulation and metabolic activity.

Lipid Accumulation Assessment (Oil Red O Staining): Cells were washed with PBS and fixed in 4% paraformaldehyde for 10 min at room temperature. Fixed cells were stained with Oil Red O for 40 min to visualize lipid droplets. The dye was subsequently eluted with isopropanol and quantified by measuring the absorbance at 490 nm.

Triglyceride Quantification: Triglycerides were extracted using a lysis buffer and quantified using a triglyceride quantification kit according to the manufacturer’s instructions. Absorbance was measured at 570 nm and concentrations were calculated against a standard curve.

Optical Density Measurements: The optical density of the dye extracted from the stained cells was measured at 490 nm using a spectrophotometer, to quantify the lipid content indicative of adipogenesis under various treatment conditions.

All experiments were conducted in triplicate. Data are expressed as mean ± standard deviation (SD). Differences between treated and control groups were analyzed using one-way ANOVA followed by Tukey’s *post hoc* test, where a *p*-value of less than 0.05 was considered statistically significant.

## 3 Results

### 3.1 Targets of active ingredients

**FIGURE 2:**
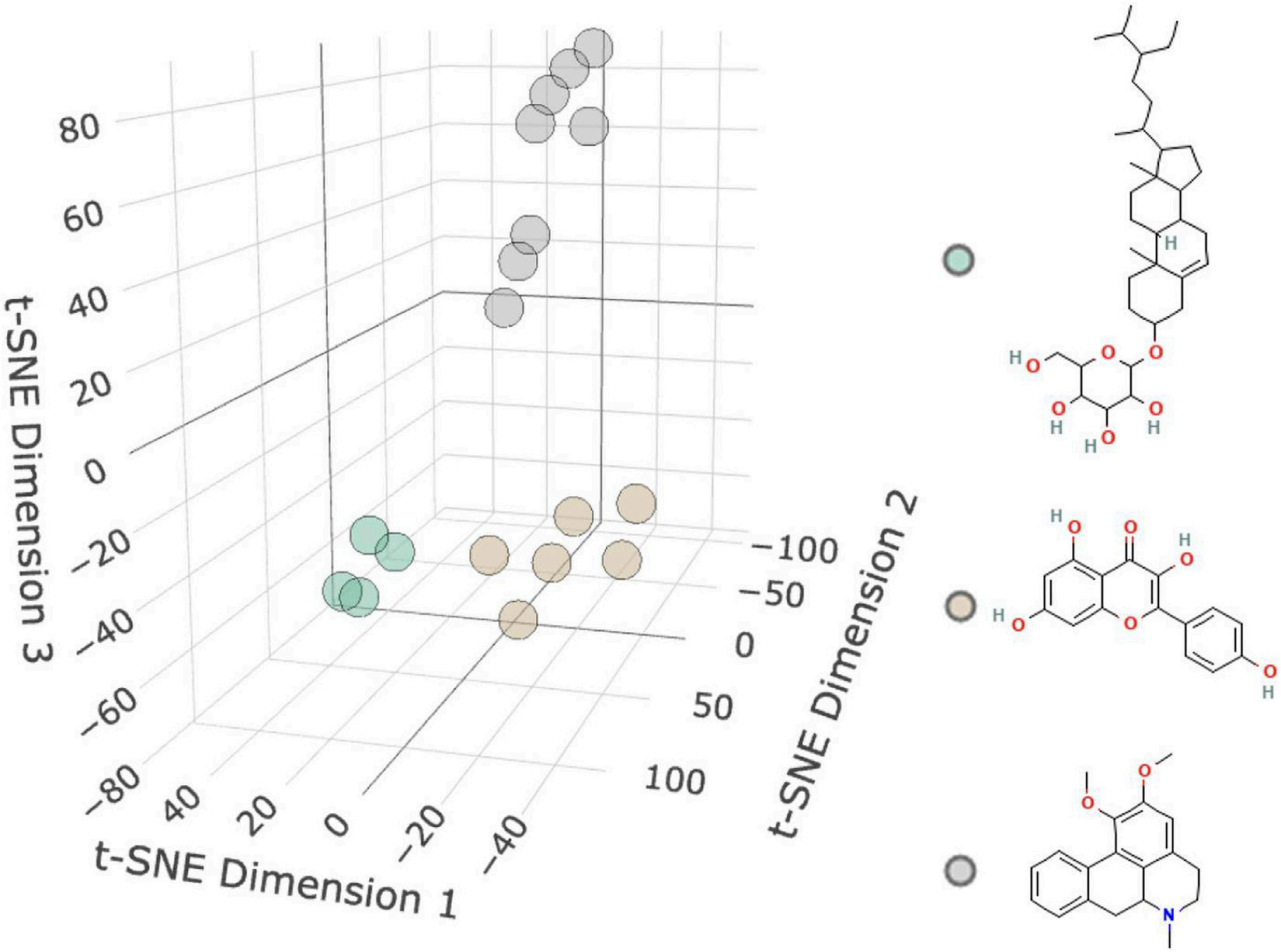
Molecular clustering diagram. The diagram shows three clusters of small molecules: the first cluster is green representing Sitogluside, the second cluster is brown representing Kaempferol, and the third cluster is gray representing Nuciferin.

As shown in Figure 2 and Table 1, 18 active molecules in *Nelumbo nucifera* leaves were clustered, resulting in three categories of molecules. The first category is represented by Sitogluside, the second category by Kaempferol, and the third category by Nuciferine.

**TABLE 1:** Results of small molecule clustering.

Smiles	V1	V2	V3	Cluster	is_representative
CC(CCC = C(C)C)C1CCC2(C1(CCC34C2CCC5C3(C4)CCC(C5(C)C)O)C)C	35.95328426	5.281447471	−60.05988609	1	FALSE
CCC(CCC(C)C1CCC2C1(CCC3C2CC = C4C3(CCC(C4)OC5C(C(C(C(O5)CO)O)O)O)C)C)C(C)C	40.93408991	23.39484502	−69.09622853	1	TRUE
COC1 = C(C=CC(=C1)C2 = C(C(=O)C3 = C(C=C(C=C3O2)O)O)O)O	−45.14556035	122.8726886	−20.70020026	2	FALSE
C1 = CC(=CC = C1C2 = C(C(=O)C3 = C(C=C(C=C3O2)O)O)O)O	−36.61972104	121.4483762	−35.90034263	2	TRUE
C1CNC2CC3 = CC = CC = C3C4 = C2C1 = CC5 = C4OCO5	10.27514164	−104.4951622	80.00591538	3	FALSE
CN1CCC2 = CC3 = C(C4 = C2C1CC5 = CC = CC = C54)OCO3	−0.424218041	−94.74319612	87.46698128	3	FALSE
C1 = CC(=C(C=C1C2 = C(C(=O)C3 = C(C=C(C=C3O2)O)O)O)O)O	−29.00356636	119.5613867	−21.19470978	2	FALSE
COC1 = C(C=C2C(NCCC2 = C1)CC3 = CC = C(C=C3)O)O	13.30287557	−51.76793009	21.76064704	3	FALSE
CN1CCC2 = CC(=C(C3 = C2C1CC4 = CC = CC = C43)OC)O	−1.523418171	−84.98220215	62.35640523	3	FALSE
C1 = C(C=C(C(=C1O)O)O)C2C(C(C3 = C(C=C(C=C3O2)O)O)O)O	−46.1954924	55.16171064	−40.39702155	2	FALSE
CCC(CCC(C)C1CCC2C1(CCC3C2CC = C4C3(CCC(C4)O)C)C)C(C)C	40.24398367	15.71520654	−52.05702476	1	FALSE
C1C(C(OC2 = CC(=CC(=C21)O)O)C3 = CC(=C(C=C3)O)O)O	−33.19215336	56.1836017	−29.13674847	2	FALSE
C1 = CC(=C(C=C1C2C(C(C3 = C(C=C(C=C3O2)O)O)O)O)O)O	−49.74114868	54.14480073	−24.83973799	2	FALSE
CN1CCC2 = CC(=C(C3 = C2C1CC4 = CC = CC = C43)OC)OC	9.217775785	−74.85162786	73.25581385	3	TRUE
CN1CCC2 = CC(=C(C=C2C1CC3 = CC = C(C=C3)O)OC)OC	7.111890518	−44.31980445	31.53113958	3	FALSE
CN1CCC2 = CC(=C(C3 = C2C1 = CC4 = CC = CC = C43)OC)OC	12.37675032	−32.78793245	9.977769123	3	FALSE
COC1 = C(C2 = C3C(CC4 = CC = CC = C42)NCCC3 = C1)OC	17.25403315	−86.55540912	62.78189844	3	FALSE
COCC1C(C(C(O1)N2C = NC3 = C(N=CN = C32)N)O)O	55.17545358	0.739200882	−75.75466988	1	FALSE

**FIGURE 3:**
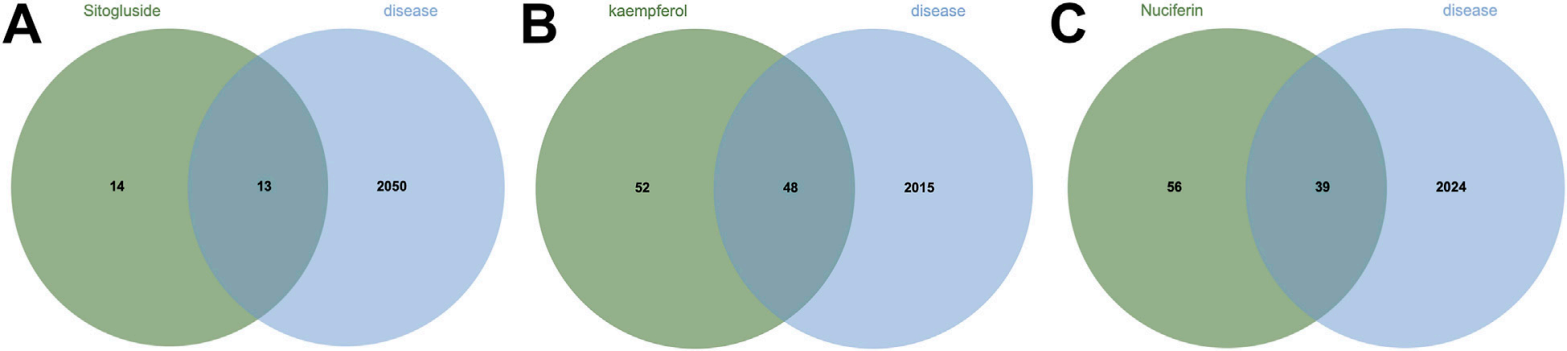
The Venn diagram illustrates the shared intersection genes between lotus leaf small molecules and obesity disease. **(A)** The number of shared intersection genes between Sitogluside and obesity disease is 13. **(B)** The number of shared genes between Kaempferol and obesity disease is 48. **(C)** The number of shared intersection genes between Nuciferin and obesity disease is 39.

**FIGURE 4:**
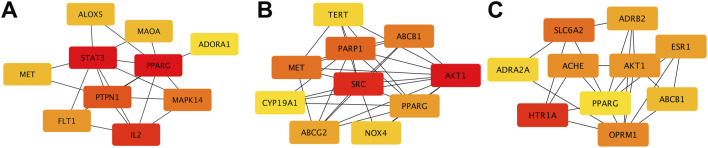
The diagram illustrates protein interactions involved in lotus leaf treatment of obesity. **(A)** Top 10 gene interactions of Sitogluside. **(B)** Top 10 gene interactions of Kaempferol. **(C)** Top 10 gene interactions of Nuciferin.

Using multiple databases, we predicted targets and intersected the results with collected obesity disease targets. As shown in Figure 3, Sitogluside intersected with 13 genes, Kaempferol with 48 genes, and Nuciferine with 39 genes. Using the Matthews Correlation Coefficient (MCC) algorithm from the cytoHubba toolkit, we identified the crucial nodes within the *Nelumbo nucifera* leaf-obesity interactome. The MCC scores, indicating the strength of connectivity, were visually represented with varying color intensities, where a deeper hue indicated higher relevance to obesity pathogenicity. We then cataloged the top 10 targets for each active small molecule, revealing key players such as PPARG, as shown in Figure 4.

### 3.2 KEGG and GO analysis

**FIGURE 5:**
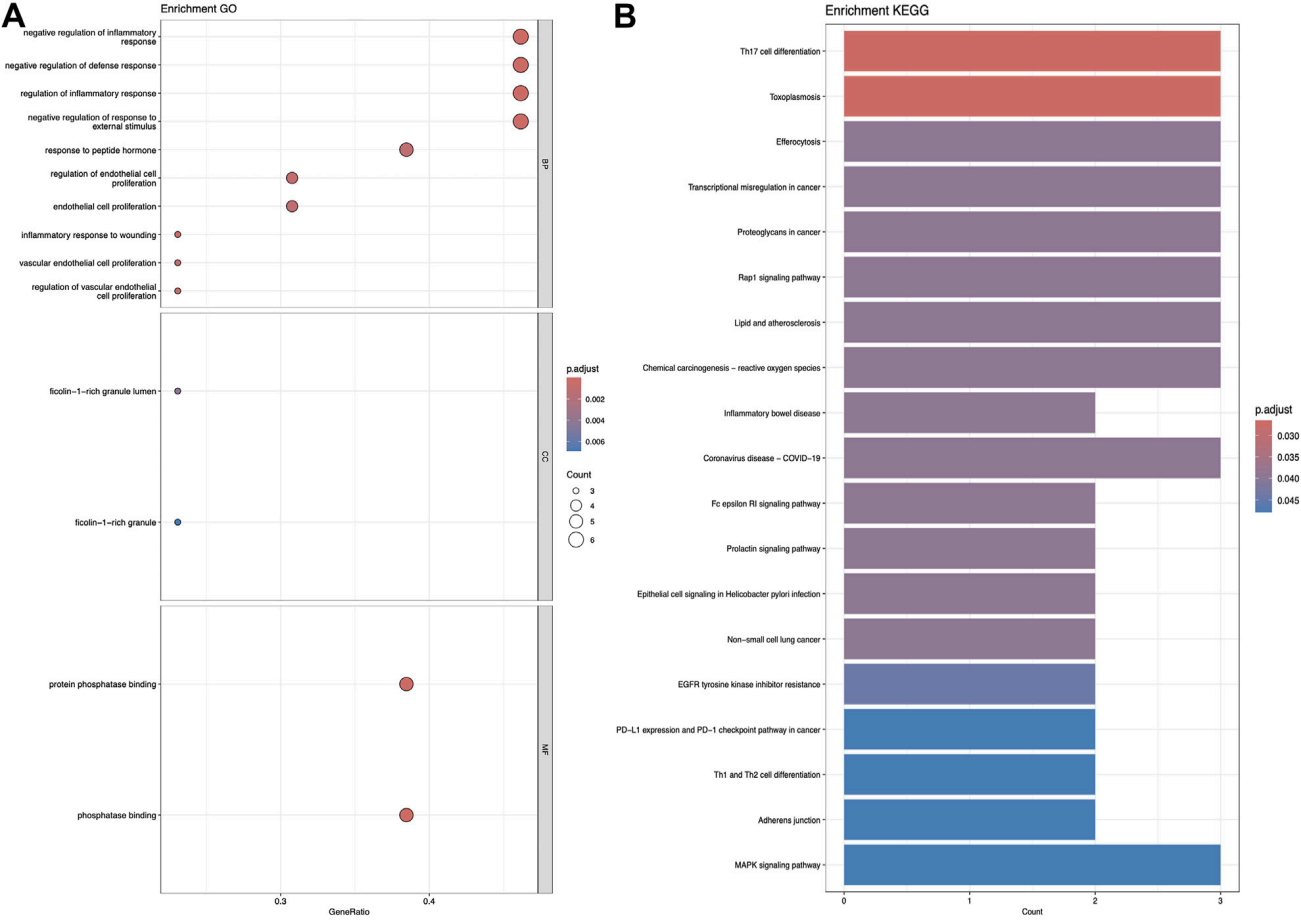
The results presented are based on the GO **(A)** and KEGG **(B)** pathway enrichment analyses of intersection genes between Sitogluside and obesity.

**FIGURE 6:**
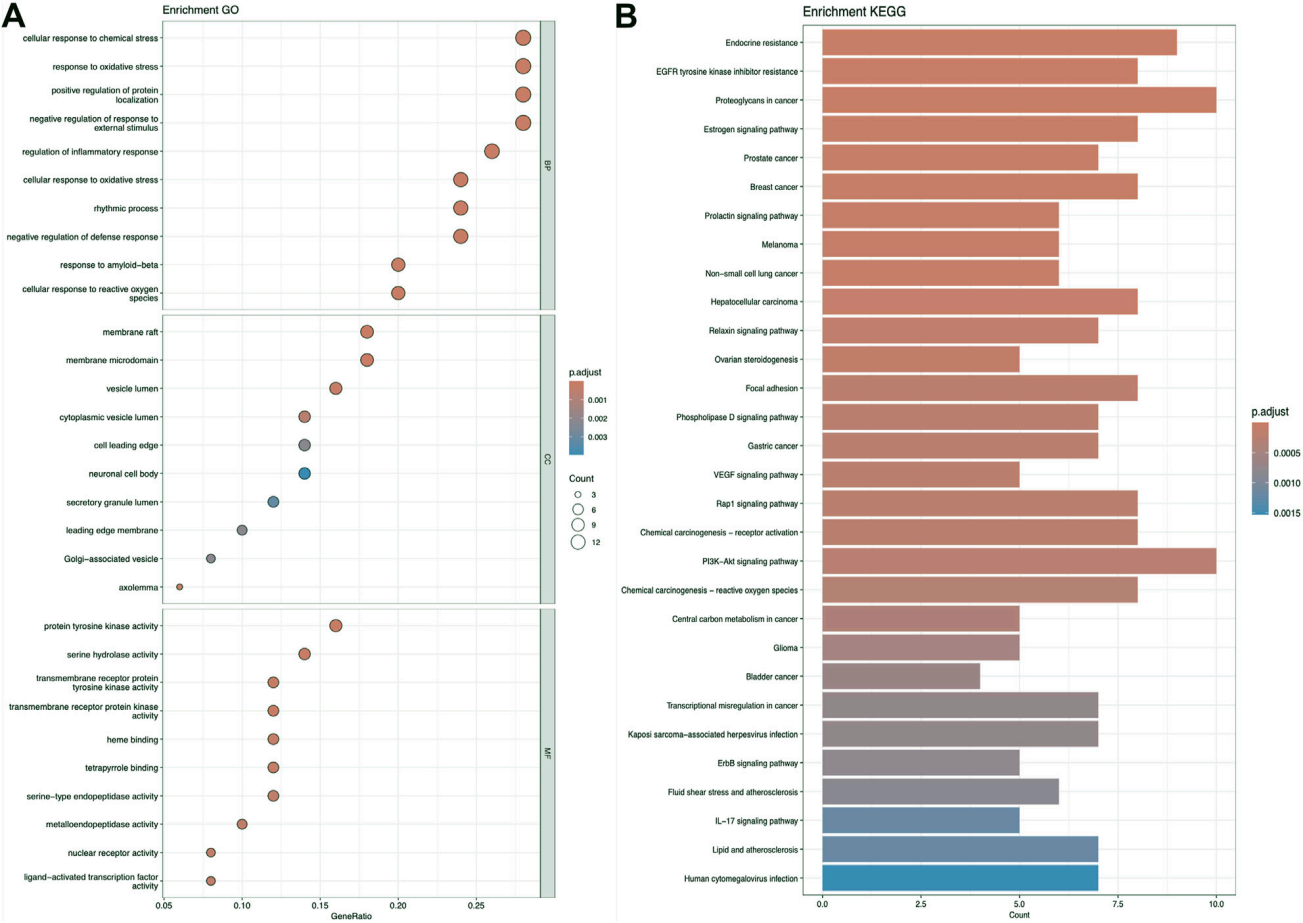
The results presented are based on the GO **(A)** and KEGG **(B)** pathway enrichment analyses of intersection genes between kaempferol and obesity.

**FIGURE 7:**
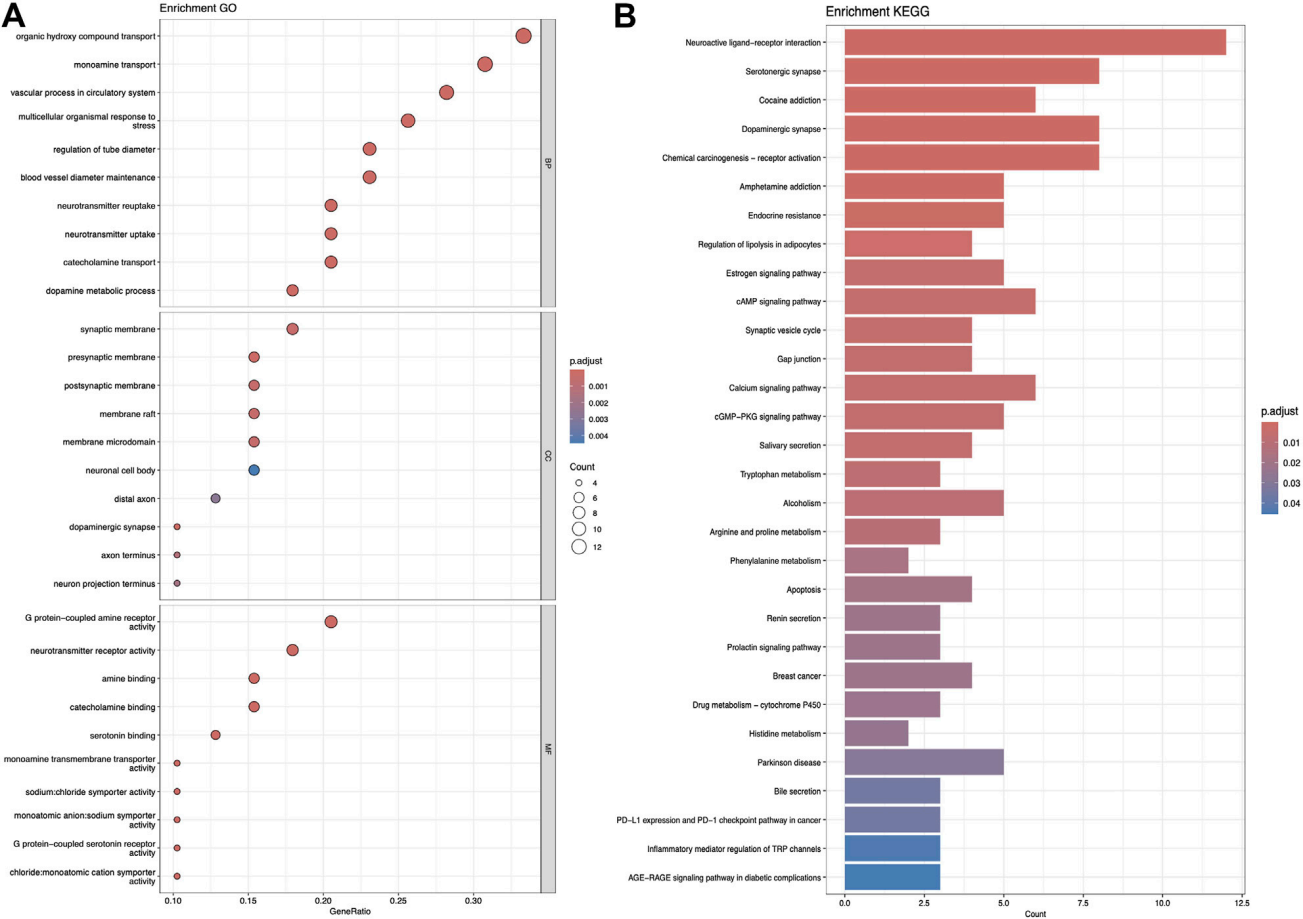
The results presented are based on the GO **(A)** and KEGG **(B)** pathway enrichment analyses of intersection genes between Nuciferin and obesity.

Figures 5–7 collectively present the results of GO and KEGG pathway enrichment analyses for genes related to *Nelumbo nucifera* leaf small molecules and obesity disease. The GO enrichment analyses (A of each figure) identify key biological processes, cellular components, and molecular functions. In the first figure, significant biological processes include cellular response to chemical stress and regulation of inflammatory response, with cellular components such as membrane rafts and neuronal cell bodies, and molecular functions focusing on protein tyrosine kinase and serine hydrolase activities. The KEGG pathway analysis highlights pathways like endocrine resistance, EGFR tyrosine kinase inhibitor resistance, and several cancer-related pathways including prostate, breast, and gastric cancer. In the second figure, enriched biological processes include organic hydroxy compound transport and vascular processes in the circulatory system, with cellular components like synaptic and presynaptic membranes and molecular functions emphasizing G protein-coupled receptor activity and neurotransmitter binding. Key KEGG pathways include neuroactive ligand-receptor interaction, serotonergic synapse, and chemical carcinogenesis, as well as addiction pathways such as cocaine and amphetamine addiction. The third figure’s GO analysis emphasizes processes like organic hydroxy compound transport and stress responses, with cellular components including membrane microdomains and neuronal cell bodies, and molecular functions like neurotransmitter receptor activity. KEGG pathway analysis reveals significant pathways such as lipid and atherosclerosis, various signaling pathways (e.g., MAPK and PI3K-Akt), and cancer-related pathways. Collectively, these analyses suggest that the interaction between *Nelumbo nucifera* leaf small molecules and obesity involves complex networks affecting inflammation, cellular signaling, and metabolic processes, with broad implications for cancer, neurological disorders, and metabolic diseases.

### 3.3 Molecular docking and machine learning screening

Due to the inclusion of PPARG as a key target for the three categories of molecules and considering the importance of PPARG in treating obesity, we performed molecular docking to screen the affinity of active small molecules in *Nelumbo nucifera* leaves for PPARG. The docking energy results are shown in Table 2. The ChemDraw (.cdx) structures of all compounds listed in Table 2 have been uploaded in the supplementary materials. Molecular docking results revealed key interactions between *Nelumbo nucifera* leaf bioactive compounds and PPARG (Table 2). Cycloartenol showed the highest binding affinity (−9.5 kcal/mol), engaging residues such as HIS351 and LYS502 through hydrophobic interactions. Sitogluside (−8.5 kcal/mol) formed hydrogen bonds with HIS351 and HIS477, and had multiple hydrophobic interactions. Isorhamnetin (−7.9 kcal/mol) formed a hydrogen bond with SER317, while kaempferol (−7.8 kcal/mol) formed one with LEU496. Both compounds also exhibited significant hydrophobic interactions. Other notable compounds, such as anonaine and remirin, showed binding affinities of −7.8 kcal/mol with distinct hydrogen and hydrophobic bonds. TThese findings highlight the potential of cycloartenol and sitogluside as promising PPARG ligands, contributing to the anti-obesity effects of *Nelumbo nucifera* leaf bioactive compounds.

**TABLE 2:** Compound_name and docking energy results.

compound_name	SMILES	Affinity (kcal/mol)	Interacting amino acid residues			
			Hydrogen bond	Alkyl	Pi-Signa	Pi-Cation
Cycloartenol	CC(CCC = C(C)C)C1CCC2(C1(CCC34C2CCC5C3(C4)CCC(C5(C)C)O)C)C	−9.5	-	HIS351 ILE500 VAL350 VAL321 LEU481 LEU497 LYS502 LYS485	-	-
Sitogluside	CCC(CCC(C)C1CCC2C1(CCC3C2CC = C4C3(CCC(C4)OC5C(C(C(C(O5)CO)O)O)O)C)C)C(C)C	−8.5	HIS351 HIS477	LYS502 LYS485 LEU481 LEU493 VAL478	-	-
isorhamnetin	COC1 = C(C=CC(=C1)C2 = C(C(=O)C3 = C(C=C(C=C3O2)O)O)O)O	−7.9	SER317	ILE500 LEU493	LEU481	HIS351
kaempferol	C1 = CC(=CC = C1C2 = C(C(=O)C3 = C(C=C(C=C3O2)O)O)O)O	−7.8	LEU496	LEU493 LEU481 LEU497 ILE500	-	HIS351
anonaine	C1CNC2CC3 = CC = CC = C3C4 = C2C1 = CC5 = C4OCO5	−7.8	ARG425 TYR348	VAL478	-	-
Remerin	CN1CCC2 = CC3 = C(C4 = C2C1CC5 = CC = CC = C54)OCO3	−7.8	-	LEU481 LYS485	LEU493	-
quercetin	C1 = CC(=C(C=C1C2 = C(C(=O)C3 = C(C=C(C=C3O2)O)O)O)O)O	−7.6	TYR355 LEU496	LEU497 LEU493 ILE500	LEU481	-
Machiline	COC1 = C(C=C2C(NCCC2 = C1)CC3 = CC = C(C=C3)O)O	−7.5	HIS351 HIS477	LEU497 ILE500	LEU481 LEU493	-
o-Nornuciferine	CN1CCC2 = CC(=C(C3 = C2C1CC4 = CC = CC = C43)OC)O	−7.4	-	ARG471	GLN472 THR475	-
leucodelphinidin	C1 = C(C=C(C(=C1O)O)O)C2C(C(C3 = C(C=C(C=C3O2)O)O)O)O	−7.4	HIS477 HIS479	LEU481 ILE500	-	HIS351
sitosterol	CCC(CCC(C)C1CCC2C1(CCC3C2CC = C4C3(CCC(C4)O)C)C)C(C)C	−7.3	TYR348	ARG471 TYR348	-	-
ent-Epicatechin	C1C(C(OC2 = CC(=CC(=C21)O)O)C3 = CC(=C(C=C3)O)O)O	−7.3		VAL478 LEU481 ILE500		HIS351
(+)-Leucocyanidin	C1 = CC(=C(C=C1C2C(C(C3 = C(C=C(C=C3O2)O)O)O)O)O)O	−7.2	-	MET491 LYS485	-	-
Nuciferin	CN1CCC2 = CC(=C(C3 = C2C1CC4 = CC = CC = C43)OC)OC	−7.1	-	ARG471	GLN472 THR475	-
Armepavine	CN1CCC2 = CC(=C(C=C2C1CC3 = CC = C(C=C3)O)OC)OC	−7	HIS351 LYS485	MET491 LYS502 LEU497	LEU493 LEU481	-
dehydronuciferine	CN1CCC2 = CC(=C(C3 = C2C1 = CC4 = CC = CC = C43)OC)OC	−6.9	-	MET491 LYS485	LEU481 LEU493	-
n-nornuciferine	COC1 = C(C2 = C3C(CC4 = CC = CC = C42)NCCC3 = C1)OC	−6.7	-	LEU485 LEU493 LEU497	LEU481	-
5′-o-methyladenosine	COCC1C(C(C(O1)N2C = NC3 = C(N=CN = C32)N)O)O	−6.3	LEU496 HIS477	LEU497	LEU481	-

**FIGURE 8:**
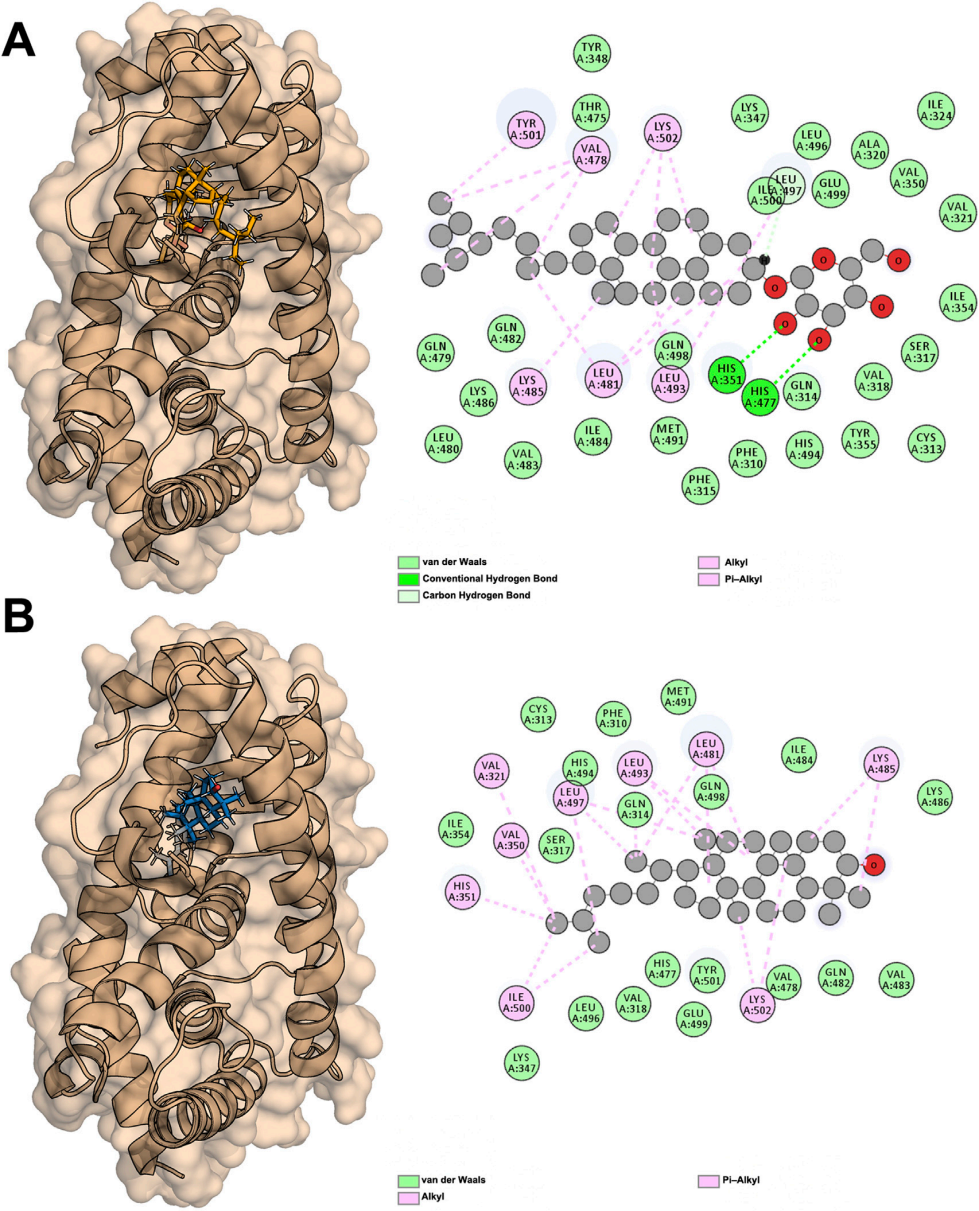
Docking results of PPARG with two active compounds from lotus Leaf. **(A)** Interaction between sitogluside and PPARG. **(B)** Interaction between cycloartenol and PPARG.

The two molecules with the highest affinity, Sitogluside (Figure 8A) and Cycloartenol (Figure 8B), both belong to the first category of molecules, with binding affinities of −8.5 kcal/mol and −9.5 kcal/mol, respectively. The docking conformations and binding sites are illustrated in Figure 8. We conducted molecular dynamics simulations on the complexes of these two molecules with the PPARG protein, as well as on the apo protein. The interaction diagram in Figure 8A highlights various residues involved in interactions: van der Waals interactions with residues such as TYR348 and LEU496; conventional hydrogen bonds with residues like HIS351 and HIS477; and alkyl interactions with residues such as VAL478 and LYS502. Similarly, the interaction diagram in Figure 8B details van der Waals interactions with residues like CYS313 and PHE310, and alkyl interactions with residues such as VAL321 and LEU497. These interactions are crucial for understanding the binding affinity and specificity of the ligands to the PPARG protein.

PPARG contains a well-defined hydrophobic binding pocket that plays a critical role in ligand recognition and stabilization. This pocket is composed of key hydrophobic residues, including LEU, VAL, and ILE, which facilitate van der Waals interactions and stabilize the binding of non-polar regions of ligands. Additionally, hydrogen bonds contribute significantly to binding specificity, with residues such as GLN487 and LYS488 forming stable hydrogen bonds with ligand functional groups. Aromatic interactions, including π-alkyl and π-π stacking with residues like TYR473 and HIS477, further enhance ligand binding by providing additional stability through π-electron interactions. These findings suggest that an effective PPARG inhibitor may ideally engage multiple interaction types, including hydrophobic, hydrogen bond, and π-interactions, to ensure both specificity and binding strength.

**FIGURE 9:**
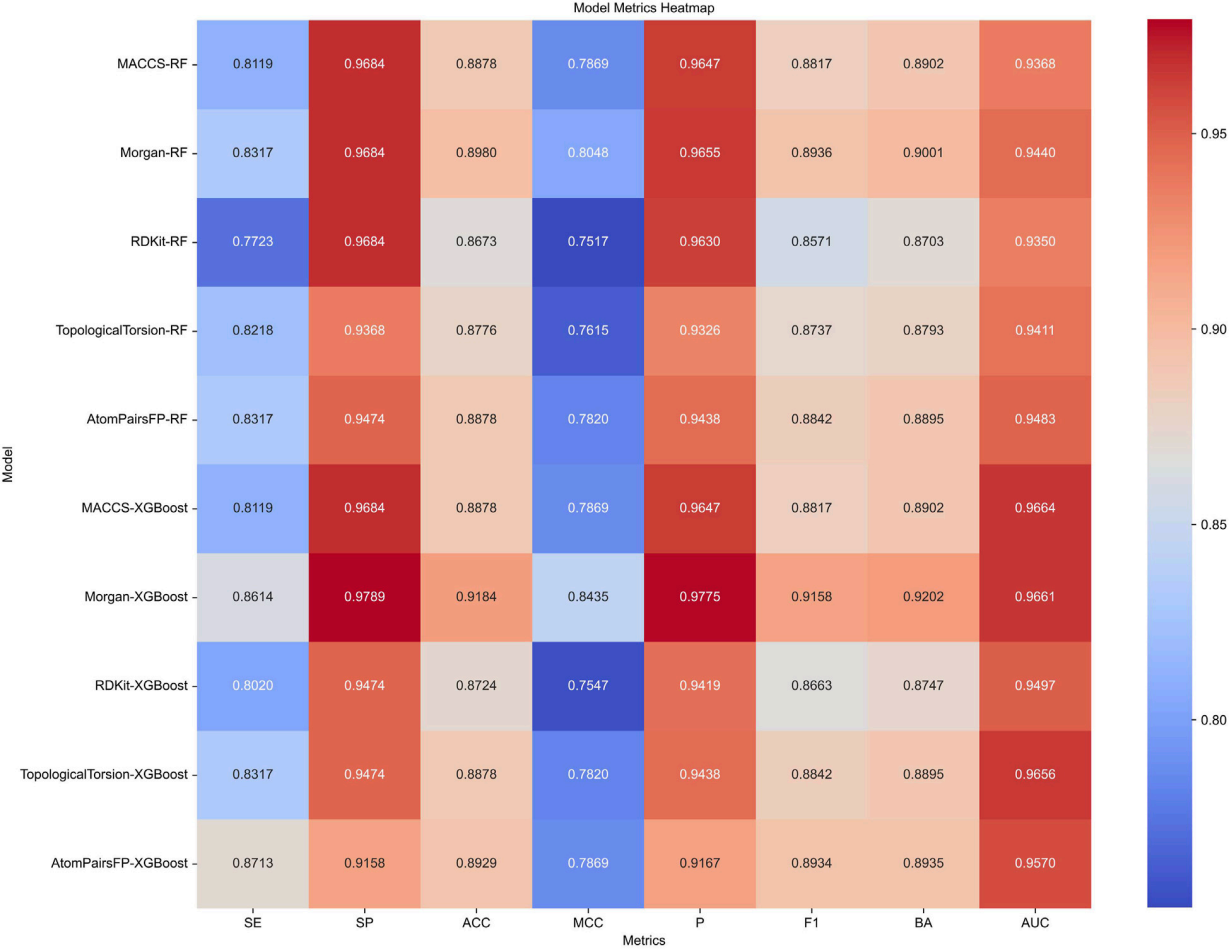
Performance metrics of Random Forest (RF) and Extreme Gradient Boosting (XGB) models using five different molecular fingerprints for PPARG inhibitor prediction.

Target prediction and reverse docking alone are insufficient for achieving biological significance, as it is challenging to distinguish between activity and inhibitory activity. To address this, we developed machine learning models for predicting PPARG inhibitors. The development of machine learning models for PPARG prediction included the use of Random Forest (RF) and Extreme Gradient Boosting (XGB) algorithms, with five molecular fingerprints (MACCS, Morgan, RDKit, Topological Torsion, AtomPairsFP) as inputs, resulting in 10 models in total. The performance of each model, evaluated using metrics such as sensitivity (SE), specificity (SP), accuracy (ACC), Matthews correlation coefficient (MCC), precision (P), F1 score (F1), balanced accuracy (BA), and area under the ROC curve (AUC), is summarized in Figure 9.

**FIGURE 10:**
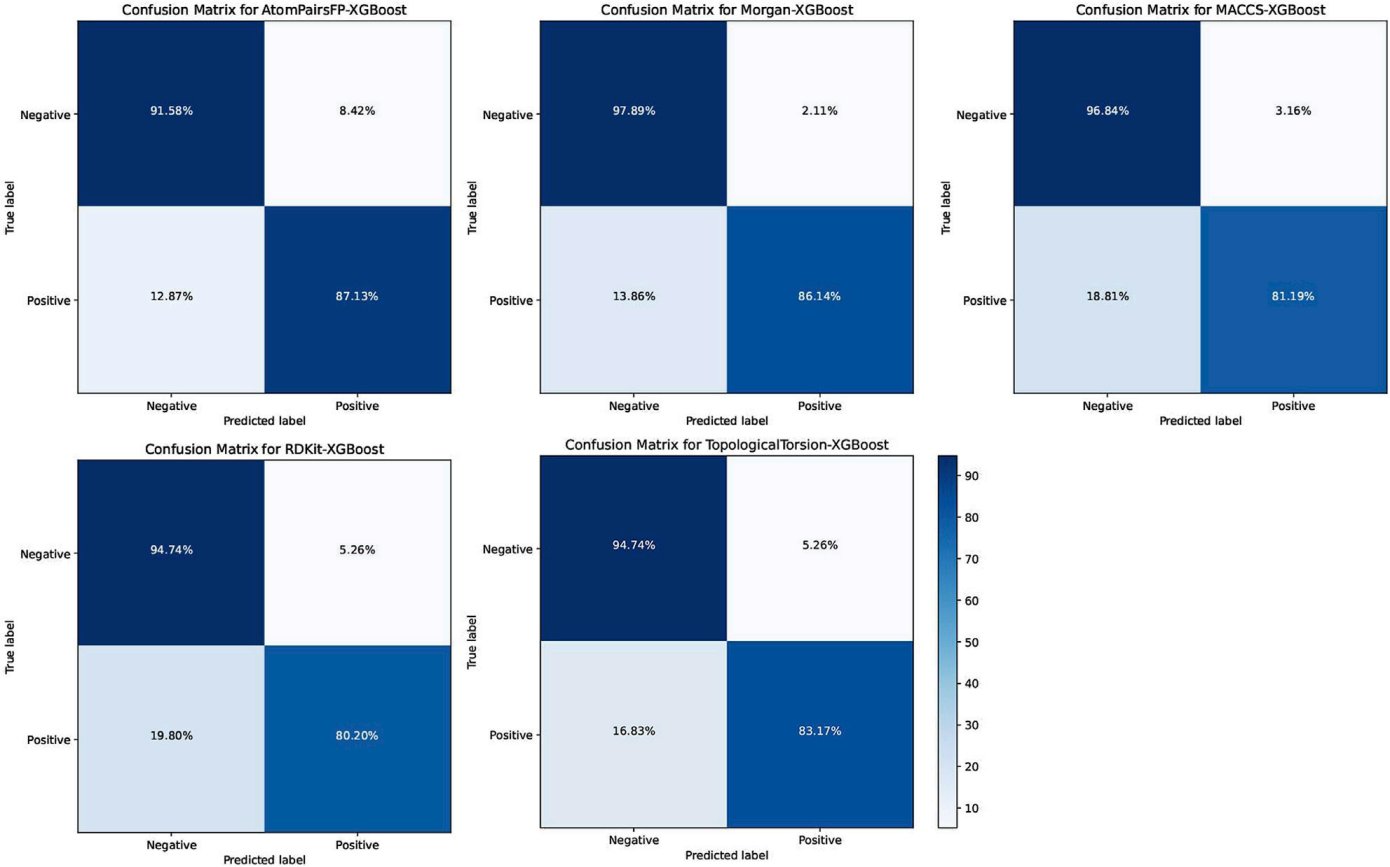
Confusion matrices for the Extreme Gradient Boosting (XGB) models using different molecular fingerprints for PPARG inhibitor prediction.

The XGB models generally performed well, with Morgan-XGBoost achieving the highest AUC (0.9661) and precision (0.9775), indicating its robustness in identifying true positive PPARG inhibitors while minimizing false positives. The confusion matrices for the XGB models (Figure 10) further demonstrate this, with Morgan-XGBoost showing a very high true negative rate (97.89%) and a reasonably high true positive rate (86.14%), suggesting that this model can reliably predict PPARG inhibitors with high specificity and good sensitivity. Other XGB models, such as AtomPairsFP-XGBoost and TopologicalTorsion-XGBoost, also showed strong performance in both specificity (91.58% and 94.74%, respectively) and sensitivity (87.13% and 83.17%, respectively). The MACCS-XGBoost model exhibited a slightly lower sensitivity (81.19%) but maintained a high specificity (96.84%), reflecting its effectiveness in filtering out false positives while being slightly more conservative in identifying true positives. The Morgan-XGBoost model, with its high precision and balanced accuracy, is particularly suited for distinguishing between active and inactive PPARG inhibitors. Using the best-performing model, Morgan-XGBoost, Cycloartenol and Sitogluside were both identified as potential PPARG inhibitors.

Several mutations, including R357A and V290M in PPARG, render it unresponsive to drugs. To mitigate this impact, we used the R357A PPARG mutant (PDB ID: 4O8F) and the V290M PPARG mutant (PDB ID: 4OJ4) and conducted molecular docking at the same site using a consistent method. The results are provided in the supplementary material. Sitogluside showed an affinity of −7.5 kcal/mol with R357A and −8.5 kcal/mol with V290M, while Cycloartenol had an affinity of −8.0 kcal/mol with R357A and −8.6 kcal/mol with V290M. It can be observed that both Sitogluside and Cycloartenol maintained high affinity in unresponsive mutants (Bharti et al., 2021).

### 3.4 Molecular dynamics simulation

**FIGURE 11:**
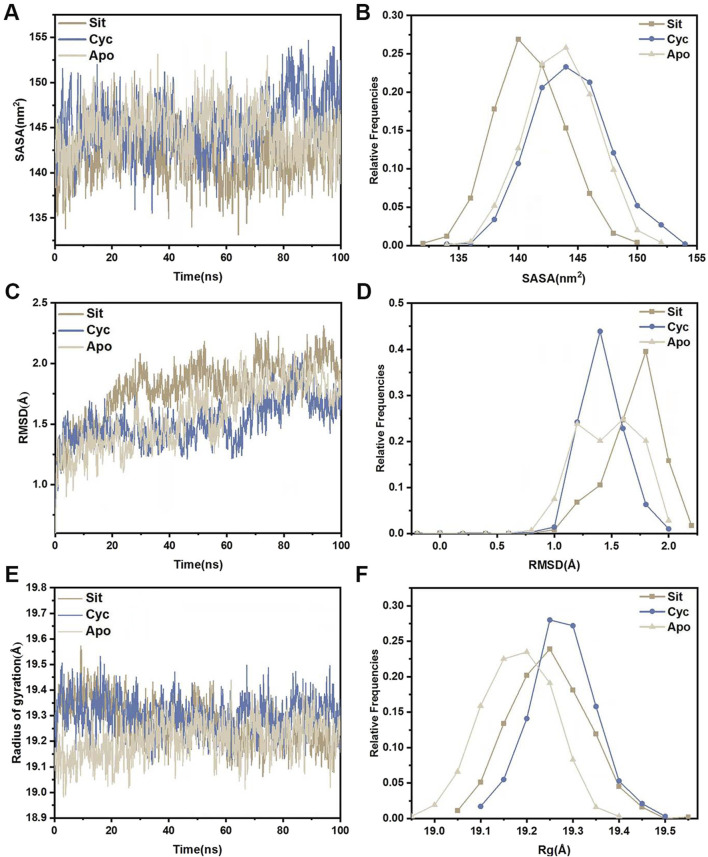
Structural stability analysis of three systems. **(A)** SASA during a 100-nanosecond molecular dynamics simulation. **(B)** Relative frequencies of SASA. **(C)** Time evolution of RMSD from their initial structures for the three systems. **(D)** Relative frequencies of RMSDs. **(E)** Radius of gyration (Rg) for three systems during a 100-nanosecond molecular dynamics simulation. **(F)** Relative frequencies of radius gyration.

*Molecular dynamics simulations were performed for the three systems (Sitogluside, Cycloartenol and Apo)*. The solvent-accessible surface area (SASA) of a protein can be used to analyze its hydrophobicity and the degree of surface exposure (Wang et al., 2023). Higher SASA values indicate greater hydrophobicity, while lower values indicate less. As shown in Figure 11A, the SASA of Sitogluside decreases after 40 ns, falling below that of the apo protein (Apo) and Cycloartenol, indicating that the conformation of the protein changes after binding with this inhibitor, resulting in increased hydrophobicity and a more closed surface. Figure 11B shows that the SASA distribution center of Sitogluside is at 140 nm^2^, while those of Apo and Cycloartenol are at 145 nm^2^, which is consistent with the trend observed in the line chart.

The root mean square deviation (RMSD) of the backbone carbon atoms relative to their initial positions is an indicator of the stability of the simulated system and reveals the deviation of the complex from its initial conformation, indicating conformational changes. Analysis of Figure 11C shows that the RMSD of Sitogluside is higher than that of the apo protein (Apo) and Cycloartenol after 20 ns, indicating greater conformational changes. As shown in Figure 11D, the RMSD distribution center of Sitogluside is at 1.7 Å, also higher than that of Apo and Cycloartenol, which aligns with the trend observed in the line chart.

The radius of gyration (Rg) reveals the compactness of the complex. Analysis of Figure 11E, F shows that the Rg of Sitogluside is smaller than that of Cycloartenol, indicating a more compact conformation after binding. However, the Rg of Sitogluside is slightly larger than that of Apo, because Apo is an apo protein without the supporting effect of a ligand.

**FIGURE 12:**
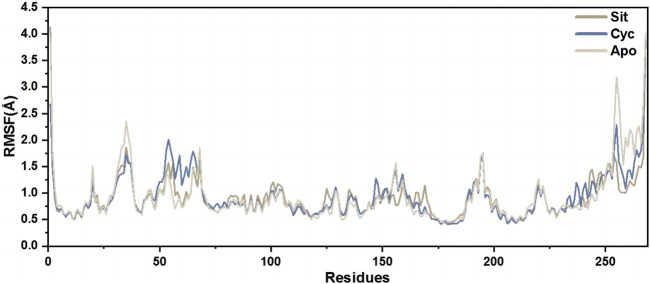
The RMSFs of the CA atoms.

The root mean square fluctuation (RMSF) of protein amino acids is used to analyze the extent of fluctuation of individual amino acids during the simulation process, revealing the flexibility changes of residues (Figure 12). The overall lower RMSF of Sitogluside indicates better stability, suggesting that Sitogluside induces a stable conformational change in PPARG. Therefore, despite having a higher RMSD value, Sitogluside maintains a relatively low RMSF.

**FIGURE 13:**
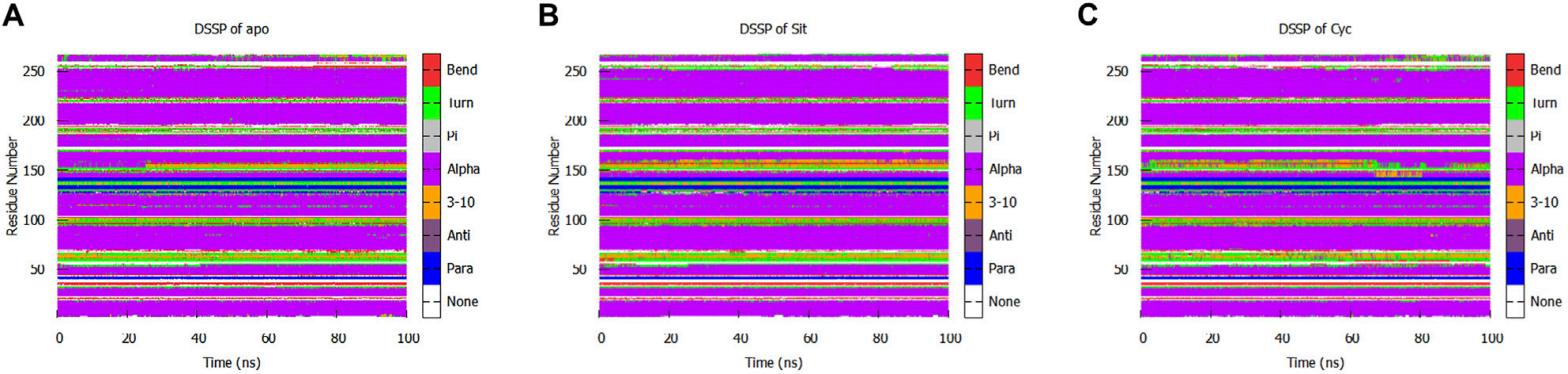
Secondary structure analysis of the protein in three systems. **(A)** Apo **(B)** Sitogluside **(C)** Cycloartenol.

We performed a protein secondary structure (DSSP) analysis and found no significant changes (Figure 13). This reveals that the influence of Sitogluside on PPARG is not closely related to secondary structure changes. Instead of affecting the conformation of alpha-helices, Sitogluside impacts the overall compactness of the protein by modulating the flexibility of loops.

**FIGURE 14:**
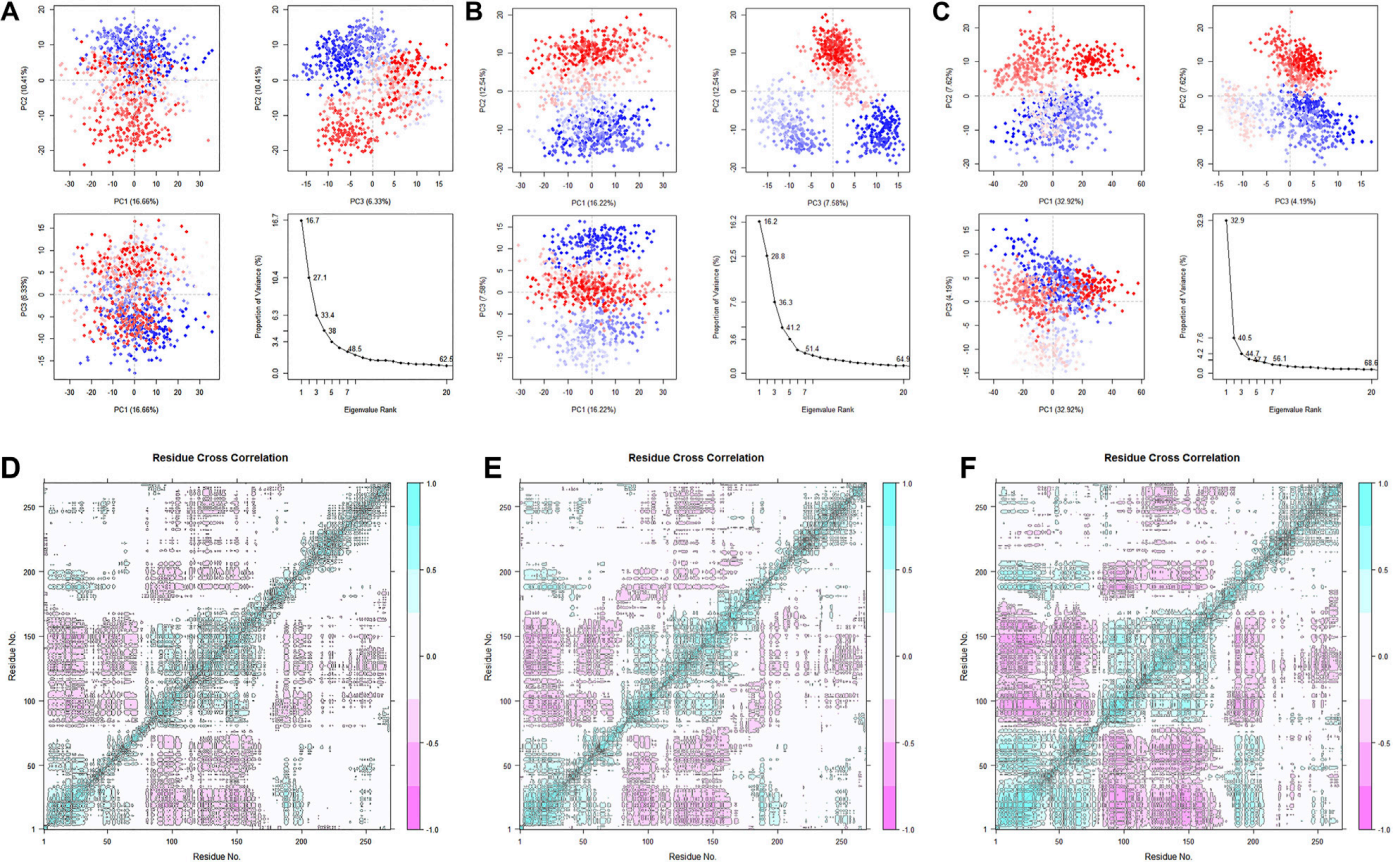
PCA analysis of the three systems. **(A)** Apo. **(B)** Sitogluside **(C)** Cycloartenol. Covariance matrix analysis of three systems. **(D)** Apo. **(E)** Sitogluside **(F)** Cycloartenol.

After studying the conformational changes, we then focused on the dynamic characteristics of the protein. The analysis of specific motion patterns, as shown by PCA (Figures 14A–C), indicates that the first two principal components of the apo group and the Cycloartenol group account for only 27.1% and 28.8% of the motion modes, with PC1 accounting for only 16.7% and 16.2%, respectively. In contrast, the first two principal components of the Sitogluside group account for 40.5%, with PC1 alone accounting for 32.9%. This reveals that the binding of Sitogluside leads to significant differences in the protein’s motion patterns.

To investigate the changes in the internal motion patterns of the protein, we calculated the dynamic cross-correlation matrix (DCCM) for each residue in the three systems (Figures 14D–F). Blue indicates positive correlated motion between related residues, while pink indicates negatively correlated motion between related residues. The diagonal correlations are relatively high because they represent the correlation of a residue with its own motion. Compared to the Apo group, the blue and pink colors in the Cycloartenol group are slightly deeper, indicating a slight increase in internal interconnectivity and a similar overall matrix shape, suggesting that its dynamic characteristics have not significantly changed.

In contrast, the Sitogluside group shows a substantial increase in internal correlation. The 1–80 region exhibits enhanced positive correlation within itself, the 80–175 region shows increased negative correlation with the 1–80 region, the 80–175 region also has increased negative correlation with the 175–200 region, and the 230–270 region shows increased positive correlation within itself. Previously uncorrelated white regions have become interrelated, visibly changing the matrix shape. Loosely packed proteins generally have lower internal motion interconnectivity, indicating that the binding of Sitogluside increases the protein’s compactness by affecting flexible regions, thereby enhancing internal stability and interconnectivity.

**FIGURE 15:**
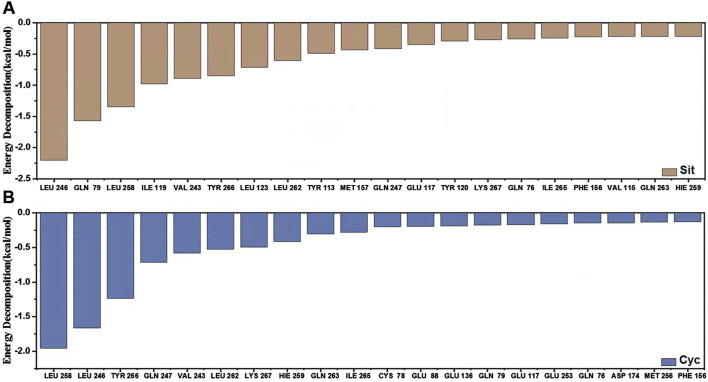
MM-PBSA energy contribution and hydrogen bonds. **(A)** Sitogluside **(B)** Cycloartenol.

Finally, we analyzed the binding energy contributions using the Molecular Mechanics Poisson-Boltzmann Surface Area (MM-PBSA) method. The binding energy are shown in Table 3, and the energy contribution graph is illustrated as Figure 15.

**TABLE 3:** The result of MM-PBSA.

System	Sitogluside	Cycloartenol
ΔEvdW	−68.81 ± 4.29	−48.29 ± 2.49
ΔEele	−4.52 ± 3.25	−2.52 ± 2.04
ΔGgas	−73.33 ± 5.19	−50.81 ± 2.99
ΔGsolv	36.84 ± 3.13	17.39 ± 1.84
ΔGtotal	−36.49 ± 3.69	−33.41 ± 2.34

The results of the MM-PBSA (Molecular Mechanics Poisson-Boltzmann Surface Area) are presented in Table 2. This method evaluates the binding free energy between the ligand and the target enzyme, providing a quantitative measure of the interaction strength. Sitogluside exhibited a binding free energy of −36.49 ± 3.69 kcal/mol, indicating a stronger binding affinity than Cycloartenol which showed a binding free energy of −33.41 ± 2.34 kcal/mol.

In summary, we identified 18 bioactive compounds from *Nelumbo nucifera* leaves with potential anti-obesity effects, focusing on their interactions with the PPARG protein. Clustering analysis classified these compounds into three distinct groups, with sitogluside, kaempferol, and nuciferine selected as representative molecules for further evaluation.

The molecular docking results revealed significant interactions between these compounds and PPARG, with sitogluside and cycloartenol displaying the highest binding affinities (−9.5 kcal/mol and −8.5 kcal/mol, respectively). Sitogluside demonstrated multiple hydrogen bonds with key residues such as HIS351 and HIS477, as well as extensive hydrophobic interactions, indicating a strong inhibitory potential. Cycloartenol exhibited similar binding strength, primarily through hydrophobic interactions involving residues such as LEU481 and VAL350.

Machine learning models, including Random Forest (RF) and Extreme Gradient Boosting (XGB), were trained to predict PPARG inhibitors based on molecular fingerprints. The XGB model using Morgan fingerprints demonstrated the best performance, achieving an AUC-ROC of 0.9661, with high precision and specificity. Sitogluside and cycloartenol were both predicted as likely PPARG inhibitors by the XGB model, validating our docking findings.

Molecular dynamics (MD) simulations were conducted for the PPARG complexes with sitogluside, cycloartenol, and the apo protein. The SASA, RMSD, and Rg analyses indicated that sitogluside binding led to a more compact and stable PPARG structure. The root mean square fluctuation (RMSF) analysis revealed that sitogluside reduced flexibility in key regions, further supporting its potential as a stabilizing ligand for PPARG. Principal component analysis (PCA) and dynamic cross-correlation matrix (DCCM) analysis highlighted significant shifts in PPARG’s motion patterns upon sitogluside binding, suggesting an allosteric effect.

Overall, the results indicate that sitogluside, followed closely by cycloartenol, exhibits strong binding and stabilizing interactions with PPARG, providing a molecular basis for their potential anti-obesity effects. These findings suggest that *Nelumbo nucifera* bioactive compounds could serve as promising candidates for natural PPARG modulators in obesity treatment.

### 3.5 Effects of Sitogluside on lipid accumulation and triglyceride levels in 3T3-L1 cells

**FIGURE 16:**
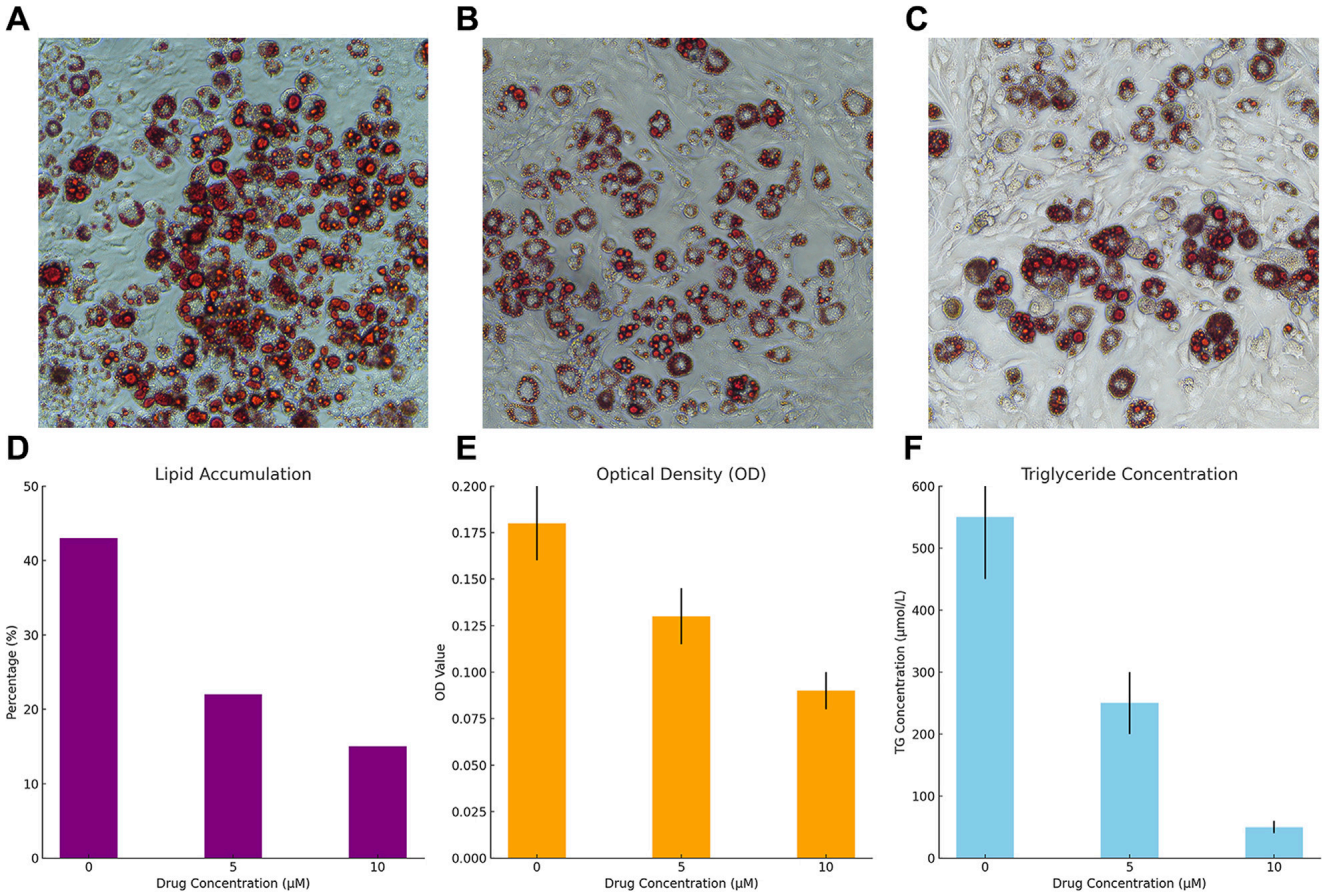
Effects of Sitogluside on Lipid Accumulation and Triglyceride Levels in 3T3-L1 Cells. **(A–C)** Representative microscopy images of 3T3-L1 cells stained with Oil Red O showing lipid accumulation at Sitogluside concentrations of 0 µM **(A)**, 5 µM **(B)**, and 10 µM **(C)**. **(D)** Graph illustrating the percentage of lipid accumulation in 3T3-L1 cells at different concentrations of Sitogluside. A clear dose-dependent decrease in lipid accumulation is observed. **(E)** Optical density (OD) at 490 nm of extracted dye from Oil Red O staining, demonstrating decreased lipid content with increasing concentrations of Sitogluside. **(F)** Triglyceride (TG) concentration measured in µmol/L, showing a significant reduction in triglyceride levels as the concentration of Sitogluside increases.

Treatment with Sitogluside significantly reduced lipid accumulation in 3T3-L1 cells in a dose-dependent manner (Figure 16A–C). The untreated cells showed a high percentage of lipid accumulation (∼43%), which decreased to ∼22% with 5 µM and further to ∼15% with 10 µM Sitogluside (Figure 16D).

Consistent with the reduction in visible lipid accumulation, the optical density measurements at 490 nm decreased with increasing Sitogluside concentrations. Cells treated with 0 µM Sitogluside exhibited an OD of approximately 0.175, which reduced to 0.125 with 5 µM and further to 0.085 with 10 µM (Figure 16E).

A similar trend was observed in the triglyceride concentrations measured in the cells. The control group displayed high triglyceride levels (approximately 500 μmol/L), which significantly dropped to about 250 μmol/L with 5 µM Sitogluside and to around 50 μmol/L with 10 µM Sitogluside treatment (Figure 16F).

These results demonstrate the efficacy of Sitogluside in modulating lipid metabolism in 3T3-L1 cells, suggesting its potential as a therapeutic agent in the treatment of obesity.

## 4 Discussion

This study investigates the potential of bioactive compounds from *Nelumbo nucifera* (lotus leaf), specifically sitogluside and cycloartenol, as inhibitors of peroxisome proliferator-activated receptor gamma (PPARG)—a nuclear receptor integral to adipogenesis and glucose metabolism. By integrating molecular docking, machine learning, and molecular dynamics (MD) simulations, we provide a comprehensive evaluation of these compounds as natural PPARG modulators. However, it is essential to contextualize our computational findings within the broader literature on PPARG inhibition, acknowledging both the strengths and limitations of our approach.

A notable strength of this study is the application of machine learning, particularly the Extreme Gradient Boosting (XGB) model with Morgan fingerprints, to enhance the accuracy and efficiency of identifying potential PPARG inhibitors. Achieving an AUC-ROC of 0.9661, our model aligns with and slightly surpasses previous studies where similar machine learning methodologies attained predictive accuracies around 0.94 for ligand classification tasks (Wu et al., 2024). This high accuracy underscores the robustness of our computational model, highlighting the growing role of artificial intelligence in accelerating the screening process for bioactive natural compounds, as evidenced in recent cheminformatics research (Wu et al., 2024; Liu et al., 2024).

Molecular dynamics simulations further enriched our understanding by revealing that sitogluside binding enhances PPARG’s structural stability, as evidenced by decreased solvent-accessible surface area (SASA), lower root mean square deviation (RMSD), and reduced radius of gyration (Rg). Previous studies have shown that ligand-induced receptor stabilization can significantly inhibit receptor activity by limiting the flexibility of key functional regions, corroborating our findings (Zhang et al., 2024). Furthermore, the observed allosteric modulation in PPARG’s motion patterns upon sitogluside binding, supported by principal component analysis (PCA) and dynamic cross-correlation matrix (DCCM) analyses, resonates with other research indicating that natural ligands may induce conformational adjustments in nuclear receptors, thereby offering a mechanism for selective inhibition.

However, this study’s reliance on computational methods also poses limitations. Molecular docking and dynamics simulations, though informative, may not capture all aspects of ligand behavior in a biological context. For instance, computational estimates of binding affinity may differ from *in vitro* or *in vivo* results due to factors like solvation effects, receptor flexibility, and simplifications in molecular models (Cataldi et al., 2021).

While sitogluside and cycloartenol exhibit high binding affinities and stabilizing effects, their pharmacokinetics and bioavailability remain unexplored. Similar studies on natural PPARG inhibitors have shown that compounds with strong computational predictions sometimes lack sufficient bioavailability or metabolic stability when tested experimentally (Stone et al., 2022). This is a critical gap that future research should address to better assess the therapeutic viability of these compounds.

In summary, sitogluside and cycloartenol emerge from our analysis as promising PPARG inhibitors, demonstrating binding affinities and stabilization effects comparable to other plant-derived PPARG modulators. However, bridging computational predictions with experimental validations remains essential to substantiate their therapeutic potential. Our findings underscore the feasibility of leveraging *Nelumbo nucifera* bioactives in anti-obesity treatments and illustrate the potential of combining machine learning with molecular modeling to streamline natural product research. Further exploration into the pharmacodynamics and clinical viability of these compounds would strengthen their candidacy as therapeutic agents.

## 5 Conclusion

This study has demonstrated the significant anti-obesity potential of *Nelumbo nucifera* Leaf Bioactive Compounds through an in-depth computational biology approach. By identifying and analyzing the active compounds in *Nelumbo nucifera* leaves, we have provided a detailed understanding of their molecular mechanisms and therapeutic effects. Clustering analysis pinpointed Sitogluside, Kaempferol, and Nuciferine as the primary active components. These molecules were found to interact with key obesity-related genes, notably PPARG, highlighting their relevance in obesity treatment.

Functional enrichment analyses using Gene Ontology (GO) and Kyoto Encyclopedia of Genes and Genomes (KEGG) pathways revealed that the active compounds influence critical biological processes and pathways, including inflammation regulation, cellular signaling, and metabolic processes. These interactions suggest a multifaceted approach by which *Nelumbo nucifera* Leaf Bioactive Compounds exert their anti-obesity effects.

Molecular docking studies demonstrated strong binding affinities of Sitogluside and Cycloartenol to PPARG, with Sitogluside showing the highest affinity. Machine learning modesl were established for PPARG inhibitors screening, Sitogluside and Cycloartenol were predicted as inhibitors.

Molecular dynamics simulations further confirmed that these interactions significantly impact the stability and conformation of the PPARG protein. Sitogluside was found to enhance the stability and compactness of PPARG, as indicated by various stability metrics such as SASA, RMSD, Rg, and RMSF.

Crucially, cellular assays demonstrated that Sitogluside significantly reduces lipid accumulation and triglyceride levels in 3T3-L1 cells, validating its functional efficacy in a biological setting.

Overall, our findings underscore the potential of *Nelumbo nucifera* Leaf Bioactive Compounds, particularly Sitogluside, as effective natural agents for obesity treatment. This study paves the way for future research and development of lotus leaf-based therapies, offering a promising alternative to conventional anti-obesity drugs.

## Data Availability

The raw data supporting the conclusions of this article will be made available by the authors, without undue reservation.
