# Supplementary figures and images for: Investigating the anti-obesity potential of Nelumbo nucifera leaf bioactive compounds through machine learning and computational biology methods
Source: Front Pharmacol. 2024 Dec 18;15:1500865. doi: 10.3389/fphar.2024.1500865 (PMC11688479; doi:10.3389/fphar.2024.1500865)

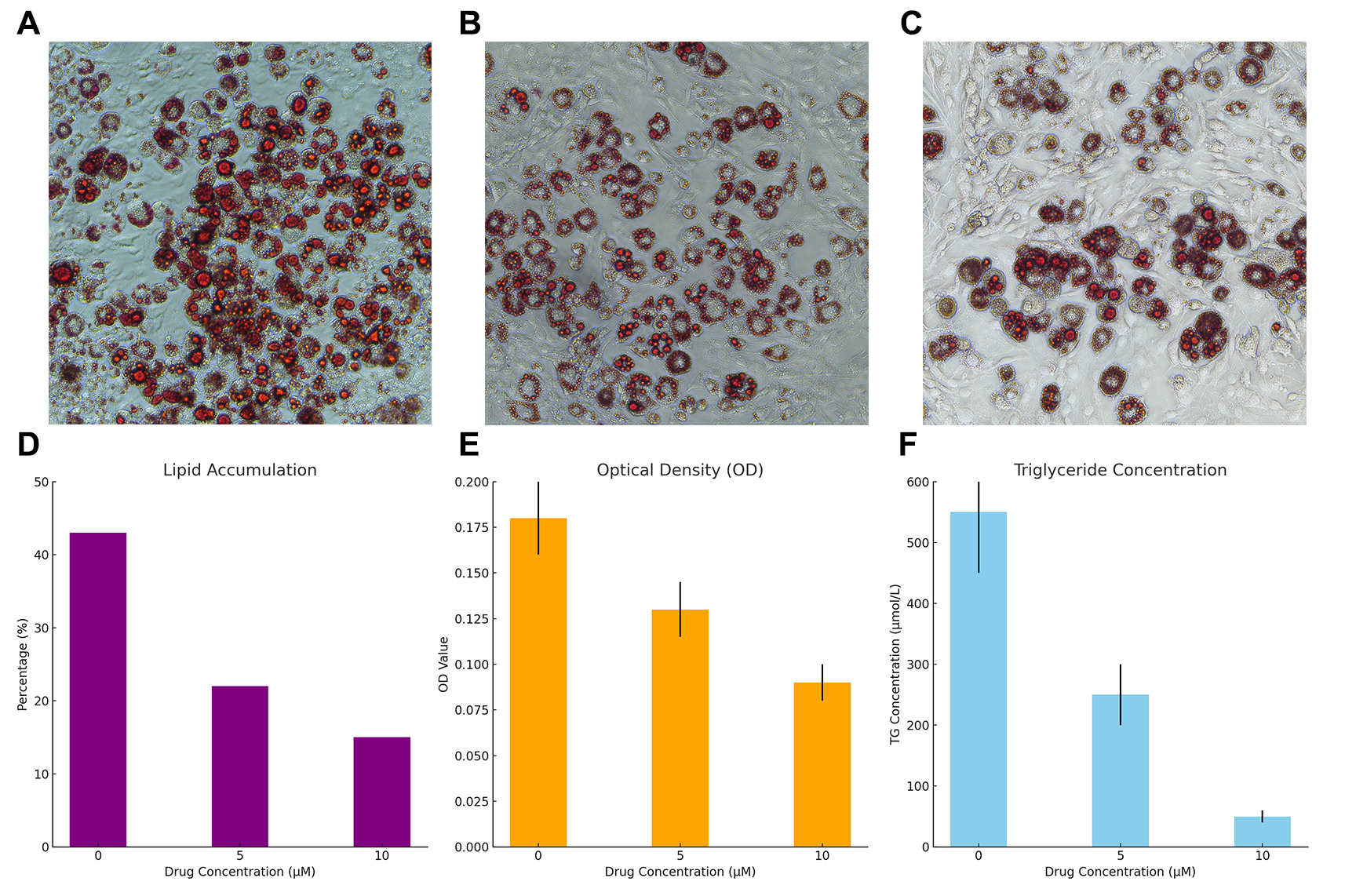

Supplement: Supplementary file 1 [file Image1.jpeg]
