# Supplementary material for: Investigating the anti-obesity potential of Nelumbo nucifera leaf bioactive compounds through machine learning and computational biology methods
Source: Front Pharmacol. 2024 Dec 18;15:1500865. doi: 10.3389/fphar.2024.1500865 (PMC11688479; doi:10.3389/fphar.2024.1500865)
Supplement: Supplementary file 2 [file DataSheet1.zip › Supplymentary.docx]

Figure S1. Molecular interactions between PPARG and its mutants with Sitogluside and Cycloartenol


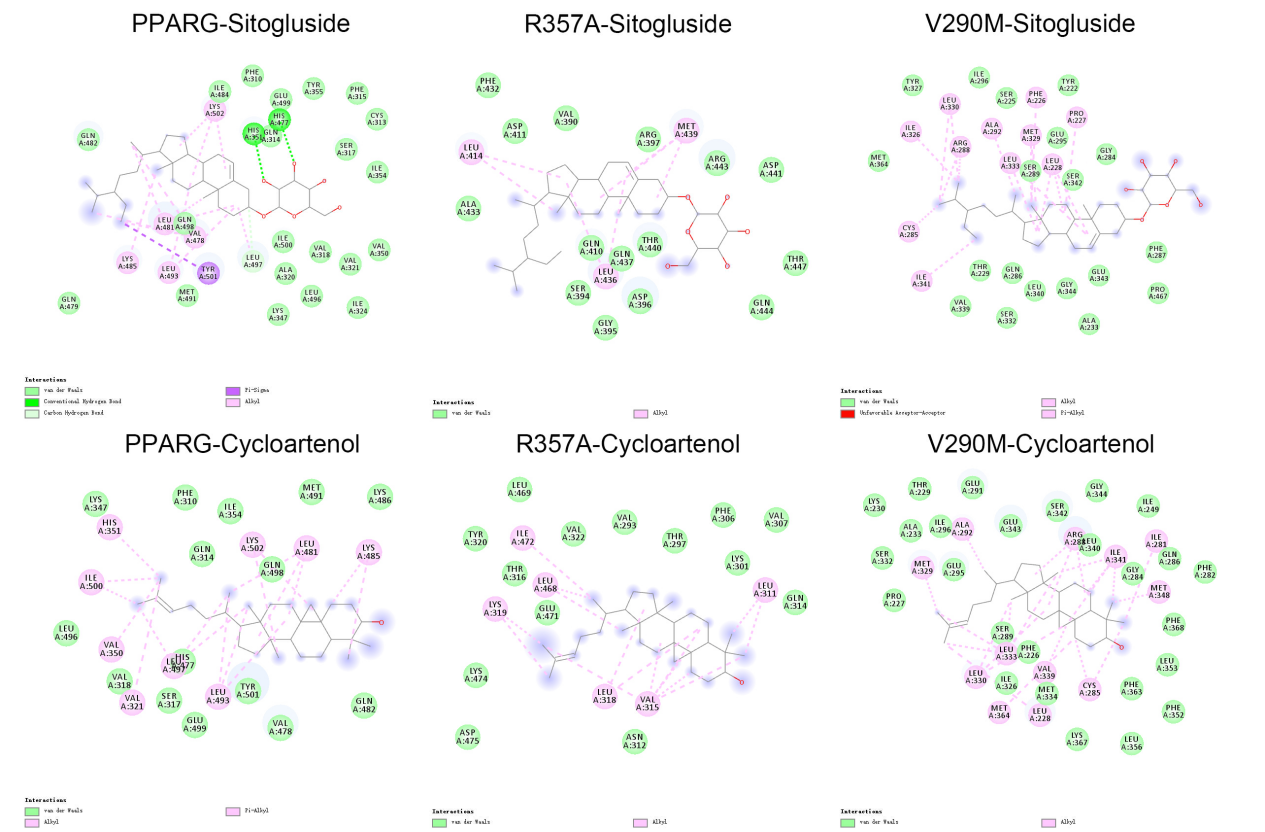


Figure S1.Binding affinity between PPARG and its mutants with Sitogluside and Cycloartenol.

| Affinity (kcal/mol) | PPARG | R357A | V290M |
| --- | --- | --- | --- |
| Sitogluside | -8.5 | -7.5 | -8.5 |
| Cycloartenol | -9.5 | -8.0 | -8.6 |
